# Validating Single-Step Genomic Predictions for Growth Rate and Disease Resistance in *Eucalyptus globulus* with Metafounders

**DOI:** 10.3390/genes16060700

**Published:** 2025-06-10

**Authors:** Milena Gonzalez, Ignacio Aguilar, Matias Bermann, Marianella Quezada, Jorge Hidalgo, Ignacy Misztal, Daniela Lourenco, Gustavo Balmelli

**Affiliations:** 1 Instituto Nacional de Investigación Agropecuaria (INIA), Tacuarembó 45000, Uruguay; mileg2014@gmail.com; 2 Instituto Nacional de Investigación Agropecuaria (INIA), Montevideo 11500, Uruguay; iaguilar@inia.org.uy; 3 Department of Animal and Dairy Science, University of Georgia, Athens, GA 30602, USA; mbermann@uga.edu (M.B.); jh37900@uga.edu (J.H.); ignacy@uga.edu (I.M.); danilino@uga.edu (D.L.); 4 Departamento de Biología Vegetal, Facultad de Agronomía, Universidad de la República, Montevideo 12900, Uruguay; mquezada@fagro.edu.uy

**Keywords:** metafounders, linear regression method, ssGBLUP, genomic selection, Eucalyptus, tree breeding

## Abstract

Background: Single-step genomic BLUP (ssGBLUP) has gained increasing interest from forest tree breeders. ssGBLUP combines phenotypic and pedigree data with marker data to enhance the prediction accuracy of estimated breeding values. However, potential errors in determining progeny relationships among open-pollinated species may result in lower accuracy of estimated breeding values. Unknown parent groups (UPG) and metafounders (MF) were developed to address missing pedigrees in a population. This study aimed to incorporate MF into ssGBLUP models to select the best parents for controlled mating and the best progenies for cloning in a tree breeding population of *Eucalyptus globulus*. Methods: Genetic groups were defined to include base individuals of similar genetic origin. Tree growth was measured as total height (TH) and diameter at breast height (DBH), while disease resistance was assessed through heteroblasty (the transition from juvenile to adult foliage: ADFO). All traits were evaluated at 14 and 21 months. Two genomic multi-trait threshold linear models were fitted, with and without MF. Also, two multi-trait threshold-linear models based on phenotypic and pedigree information (ABLUP) were used to evaluate the increase in accuracy when adding genomic information to the model. To test the quality of models by cross-validation, the linear regression method (LR) was used. Results: The LR statistics indicated that the ssGBLUP models without MF performed better, as the inclusion of MF increased the bias of predictions. The ssGBLUP accuracy for both validations ranged from 0.42 to 0.68. Conclusions: The best model to select parents for controlled matings and individuals for cloning is ssGBLUP without MF.

## 1. Introduction

Forest tree breeding has successfully delivered genetically improved material for multiple traits through recurrent selection, mating, and testing [1]. However, long breeding cycles, late flowering, variable juvenile-mature correlations, emerging pests, diseases, and climate and market changes pose significant challenges [2]. Open-pollinated populations are commonly used in tree breeding due to their simplicity and cost-effectiveness [3]. This mating design is one of the most frequently employed, as its simplicity and low cost enable the evaluation of the genetic merit of numerous individuals without requiring controlled mating [4]. Nevertheless, missing pedigree information in such populations complicates accurate genetic evaluation and selection [5]. This challenge arises because open pollination involves random mating with unknown paternal contributors [6].

Genomic selection (GS) is expected to bring a paradigm shift in tree breeding by enhancing its speed and efficiency [7]. By simultaneously fitting all genome-wide markers, GS can capture much of the “missing heritability” associated with complex traits [8]. Single-step genomic best linear unbiased prediction (ssGBLUP) is a widely adopted method in animal breeding that integrates pedigree, phenotypic, and genomic information into a unified evaluation framework [9,10,11,12]. In GS, an estimation or “training” population of several hundred or thousands of individuals is genotyped using a genome-wide marker panel and phenotyped for target traits of interest [13]. From these datasets, prediction models are developed and validated in a “validation” set, employing appropriate methods to prevent overfitting [14].

In *Eucalyptus* species, several studies have demonstrated that using genomic tools to estimate relationships improves genetic evaluation accuracy for traits like growth, wood density, and disease resistance [8,15,16]. However, missing pedigree information can introduce biases and inflate genomic estimated breeding values (GEBVs) in ssGBLUP [17]. This issue arises due to pedigree-based and genomic relationship matrices incompatibility, leading to inaccurate genetic evaluations [18]. Unknown parent groups (UPG) were developed to account for missing pedigrees in a population [19,20]; assigning UPG to missing parents helps mitigate biases in genetic evaluations [21,22]. Nevertheless, the effectiveness of UPG in ssGBLUP depends on the availability of sufficient data; inadequate data can lead to biased genetic trends and underestimated UPG effects [22,23]. The UPG approach still assumes that the base populations are unrelated, which is often not true [22,24]. In practice, the pedigrees are incomplete, and individuals with information descend from different base populations [25].

Metafounders (MF) also model missing pedigrees by assuming that an ancestral population is represented as a single pseudo-individual (a metafounder) with a particular self-relationship (a measure of homozygosity) that represents a pool of gametes [26]. The MF approach generalizes UPG by assuming them as inbred and related by a covariance matrix called Γ [26,27,28]. This method has shown promise in reducing biases, especially in multibreed or crossbred evaluations, provided the dataset allows for estimating Γ reasonably well [26,29]. When MF are used, individuals from different breeds can be related through pedigree, improving the compatibility between genomic and pedigree relationships [24,30]. In the MF approach, the goal is to adjust the pedigree-based relationship matrix $\left(A\right)$ to achieve compatibility with a genomic relationship matrix $\left(G\right)$ constructed with an assumed allelic frequency of 0.5 [26]. The vector of additive genetic effects $\left(u\right)$ in this model encompasses genotyped and non-genotyped individuals and metafounders.

*Eucalyptus globulus* is a globally significant species in forestry, particularly valued for its rapid growth and high-quality wood for pulp and paper production [31]. In recent years, *E. globulus* plantations have been severely affected by the disease caused by *Teratosphaeria nubilosa* [32], significantly damaging young plantations [33]. *T. nubilosa* is a serious pathogen affecting many *Eucalyptus* spp. leaves [32,34]. This pathogen primarily infects juvenile and intermediate foliage, causing severe leaf spotting, necrosis, defoliation, and shoot blight [35]. In *E. globulus*, an early heteroblastic transition has been observed as a natural defense mechanism [36]. Adult leaves demonstrate significantly lower susceptibility to *T. nubilosa* than juvenile foliage [32]. In regions where *T. nubilosa* is prevalent, alternative *Eucalyptus* spp. or hybrids with inherent resistance are being explored [37]. GS models offer an effective tool to predict genetic potential for disease resistance by analyzing the entire genome, enabling breeders to select individuals with superior resistance traits [38,39].

In 2017, the National Agricultural Research Institute (INIA) in Uruguay initiated a program to develop GS models to predict growth and disease resistance in *E. globulus*. The first approach, carried out by Quezada et al. [40] using an open-pollinated population, achieved high accuracies in estimating genetic parameters and improving the prediction of breeding values for ranking and selecting individuals. To further advance the effective incorporation of GS into the breeding program for *E. globulus*, the main objective of this study was to compare the GS using in a multi-trait threshold linear models with and without MF, to select the best parents for controlled mating and the best progenies for cloning.

## 2. Materials and Methods

### 2.1. Study Population

The study population is derived from the *E. globulus* tree improvement program coordinated by INIA Uruguay. It includes both parent trees and their progeny. Parent material originated from two seed orchards established in 1996 (first generation) and 2002 (second generation). Progeny were evaluated in six trials located in southeastern Uruguay (Lavalleja and Rocha), regions commonly used for *E. globulus* plantations. The first trial, established in 2011, consists of 3853 individuals from 194 open-pollinated (OP) half-sib families, primarily from the first-generation orchard (see Quezada et al. [40]). The other five trials, established between 2015 and 2019, comprise 6051 individuals from 137 full-sib families generated through controlled matings (CM) from the second-generation orchard (Table 1).

Tree growth was assessed by measuring total height (TH) with a hypsometer (Vertex IV HS 102, Haglöf Sweden, Långsele, Sweden) and diameter at breast height (DBH) using a diameter tape at 14 and 21 months, respectively. In addition, the precocity of the transition from juvenile to adult foliage (ADFO) was evaluated at the same time points. ADFO was visually estimated as the percentage of the crown exhibiting adult foliage, using a scale in 10% increments. Due to an uneven distribution of observations across these intervals, values were consolidated into three categories for analysis: 1 = no adult foliage, 2 = up to 50%, and 3 = more than 50%. ADFO was analyzed as a categorical trait, while TH and DBH were treated as quantitative traits. At 14 months, most individuals exhibited only juvenile foliage; by 21 months, the majority showed up to 50% adult foliage, with relatively few exceeding this threshold (Table 2).

The genotypic dataset comprised 2,409 individuals, including both parents and progeny from OP and CM families (Table 1). Genotyping was performed using the *Eucalyptus* EUchip60K SNP chip [41] by GeneSeek Inc. (Lincoln, NE, USA). Quality control filtering was conducted with PREGSF90 version 1.26 [42], retaining markers with a call rate > 0.95 and a minor allele frequency (MAF) > 0.05, as well as individuals with a call rate > 0.80. Following filtering, 15,821 SNP markers and 2359 individuals remained for downstream analyses. Pedigree correction was carried out using SEEKPARENTF90 version 1.56 from the BLUPF90 software suite version 2.6 [43].

### 2.2. Multi-Trait Threshold Linear ssGBLUP Model

Four multi-trait threshold linear models were compared to check the effect of including MF with and without genomic information. The matrix notation is specified by Equation (1):

(1) $$\begin{array}{c} y_{1}\\ y_{2}\\ l_{3}\\ l_{4} \end{array}=\left[\begin{array}{cccc} X_{1} & O & O & O\\ O & X_{2} & O & O\\ O & O & X_{3} & O\\ O & O & O & X_{4} \end{array}\right]\begin{array}{c} b_{1}\\ b_{2}\\ b_{3}\\ b_{4} \end{array}+\left[\begin{array}{cccc} W_{1} & O & O & O\\ O & W_{2} & O & O\\ O & O & W_{3} & O\\ O & O & O & W_{4} \end{array}\right]\begin{array}{c} p_{1}\\ p_{2}\\ p_{3}\\ p_{4} \end{array}+\left[\begin{array}{cccc} Z_{1} & O & O & O\\ O & Z_{2} & O & O\\ O & O & Z_{3} & O\\ O & O & O & Z_{4} \end{array}\right]\begin{array}{c} u_{1}\\ u_{2}\\ u_{3}\\ u_{4} \end{array}+\begin{array}{c} e_{1}\\ e_{2}\\ e_{3}\\ e_{4} \end{array}$$

where y_1_ and y_2_ is the vector of observed phenotypes for TH and DBH, l_3_ and l_4_ is a vector of unobserved liabilities for ADFO at 14 months and ADFO at 21 months, b is the vector of fixed effects, including the overall mean of sites and blocks (within site) with incidence matrix X; p is the vector of random plot effects with incidence matrix W; u is the vector of random genetic additive effects of the individual trees, with incidence matrix Z; and e is the vector of random residuals. Under the assumed threshold model, the observed phenotypes for individual i is dictated by its liability (l_3_ and l_4_), with the following relationship: if l_3_ and l_4_ exceeds an unknown fixed threshold (t), then y_3_ and y_4_ = 1 (no adult foliage), y_3_ and y_4_ = 2 (up to 50% adult foliage) and y_3_ and y_4_ = 3 (more than 50% adult foliage). We assumed that liability was normally distributed with mean vector θ and variance $\sigma_{e}^{2}$ as specified by Equation (2):

(2) $$l_{3}\;and\;l_{4}|\theta ,\sigma_{e}^{2}\;\sim \;N\left(S\theta ,I\sigma_{e}^{2}\right);\;\sigma_{e}^{2}$$

where θ′ = (b′, p′, u′) is a vector of systematic and random effects, and S is an incidence matrix (containing the X, W and Z) for linking θ to the phenotypic records. The FOAD response at 14 months, given the liability and the threshold, can be expressed as Equation (3):

(3) $$\begin{array}{c} p\left(y_{3}|l_{3},t\right)=\Pi_{i=1}^{n}[I\left(l_{3}\leq t_{1}\right)I\left(y_{3}=1\right)\\ +I\left(t_{1}<l_{3}\leq t_{2}\right)I\left(y_{3}=2\right)\\ +I\left(t_{2}<l_{3}\right)I\left(y_{3}=3\right)] \end{array}$$

where t_1_ and t_2_ are thresholds that define the three categories of response and I is an indicator function that takes value 1 if the condition specified is true, otherwise the value is 0. The FOAD response at 21 months, given the liability and the threshold, is expressed in the same way.

The models differed in the relationship matrices used for the variance of additive genetic effects. The first genomic model assumed $Var\left(u\right)=H⊗\Sigma$ (Aguilar et al., 2010 [11]), whereas the second included metafounders and assumed that $Var\left(u\right)=H^{\Gamma }⊗\Sigma$. Similarly, the first model assumed $Var\left(u\right)=A⊗\Sigma$, and the other assumed $Var\left(u\right)=A^{\Gamma }⊗\Sigma$.

The (co)variance structure was defined to include additive genetic, plot, and residual variances for each trait, incorporating both pedigree and genomic information, as shown in (4):

(4) $$Var\left[\begin{array}{c} p\\ u\\ e \end{array}\right]=\left[\begin{array}{ccc} I⊗P_{0} & 0 & 0\\ 0 & H⊗\Sigma /H^{\Gamma }⊗\Sigma /A⊗\Sigma /A^{\Gamma }⊗\Sigma & 0\\ 0 & 0 & I⊗R_{0} \end{array}\right]$$

where Σ, $P_{0}$ and $R_{0}$ are 4 × 4 matrices of (co)variances for additive genetic, plot effects, and residual; **I** is the identity matrix, a square matrix with 1 on the diagonal and 0 on the off-diagonal, H is the realized relationship matrix that combines pedigree and genomic relationships. $H^{\Gamma }$ and $A^{\Gamma }$ are the relationship matrices augmented with metafounders.

The inverse of H $\left(H^{-1}\right)$ was derived by Aguilar et al. [11] as defined by Equation (5):

(5) $$H^{-1}=A^{-1}+\left[\begin{array}{cc} 0 & 0\\ 0 & G^{-1}-A_{22}^{-1} \end{array}\right]$$

where $A^{-1}$ and $A_{22}^{-1}$ are the inverse of the pedigree relationship matrices for all individuals and only for the genotyped individuals, respectively, and $G^{-1}$ is the inverse of the genomic relationship matrix, calculated according to VanRaden [44]. The MF were defined based on the origin and breeding generations of the seeds: Australia, Uruguay, and two seed orchards (established in 1996 and 2002).

### 2.3. Variance Components

Variance components and (G)EBVs for each model were estimated using GIBBSF90+ [45] version 3.2. For variance components, the Gibbs sampling process comprised 300,000 samples, and one every 50th sample was stored. After discarding the first 10,000 samples as burn-in, posterior means were calculated. For (G)EBV, variance components were fixed to the posterior means, and 30,000 samples were drawn. Variance components were calculated with genomic information for four models. Posterior means of (G)EBV were used in the validation process.

The narrow sense heritability for all traits was estimated for the models without MF (ssGBLUP and ABLUP), as follows in (6):

(6) $$h^{2}=\frac{\sigma_{a}^{2}}{\sigma_{a}^{2}+\sigma_{p}^{2}+\sigma_{e}^{2}}$$

where $\sigma_{a}^{2}$ is the additive genetic variance, $\sigma_{p}^{2}$ is the plot variance, and $\sigma_{e}^{2}\;$ is the residual variance.

The genetic correlations for all traits were estimated for the models without MF (ssGBLUP and ABLUP) as follows in (7):

(7) $${rg}_{ij}=\frac{\sigma_{ij}}{\sqrt{\sigma_{i}^{2}+\sigma_{j}^{2}}}$$

where $\sigma_{i}^{2}$ and $\sigma_{j}^{2}$ are the additive genetic variances for the *i*th and *j*th traits, respectively, and $\sigma_{ij}$ is their covariance.

Non-additive genetic effects and genotype-by-environment (G × E) interactions were excluded from narrow-sense heritability estimates, as only additive genetic variance contributes directly to predictable evolutionary change. In crosses within the same species, particularly among individuals from genetically similar populations, genetic divergence is limited, and most of the genetic variation is expected to be additive. G × E interactions were also omitted from the analysis because selection was aimed at a single breeding zone (southeastern Uruguay), where environmental conditions are relatively uniform. The focus was on identifying genotypes with superior performance in this specific context.

All analyses were carried out using BLUPF90 family software version 2.6 [46] and R package ggplot2 [47] version 3.4.2.

### 2.4. Model Validation Using LR

To complement cross-validation approaches, we followed a semiparametric procedure based on the classical theory of genetic evaluation, the linear regression method (LR) [48]. They proposed to test the quality of evaluation methods using cross-validation statistics based on successively estimated breeding values (EBV) from a set of “focal” individuals. These “focal” individuals can be the whole population or a group of individuals of interest, such as candidates for selection. The focal individuals have no phenotype in the partial dataset (p) but have a phenotype in the whole dataset (w). The whole dataset contains all phenotypes, and the partial dataset contains phenotypes of a smaller group of individuals. Then, validation statistics compare EBV obtained from the “whole” dataset versus EBV obtained in the “partial” dataset. According to Legarra and Reverter [48], the correlation between the whole and partial datasets is a measure of the expected magnitude of the change in accuracy with increasing information, as specified by Equation (8):

(8) $$\rho_{wp}=\frac{cov\left({\hat{u}}_{w},{\hat{u}}_{p}\right)}{\sqrt{var\left({\hat{u}}_{w}\right)\;var\left({\hat{u}}_{p}\right)}}$$

where ${\hat{u}}_{w}$ and ${\hat{u}}_{p}$ are the GEBV estimated with the whole and partial data, respectively. The bias is defined as the difference in their means as follows in (9):

(9) $$\mu_{wp}=\bar{{\hat{u}}_{p}}-\bar{{\hat{u}}_{w}}$$

and the dispersion as the slope of the regression of ${\hat{u}}_{w}$ on ${\hat{u}}_{p}$ as follows in (10):

(10) $$b_{wp}=\frac{cov\left({\hat{u}}_{w},{\hat{u}}_{p}\right)}{var\left({\hat{u}}_{p}\right)}$$

The bias has an expected value of 0 if the evaluation is unbiased. The dispersion has an expected value of 1 if there is no over/under dispersion. The accuracy of GEBV in the partial dataset is calculated as follows in (11):

(11) $$\rho_{cov\left(w,p\right)}=\sqrt{\frac{cov\;({\hat{u}}_{w},{\hat{u}}_{p})}{\left(1-\bar{F}\right)\;{\sigma_{u}}^{2}}}$$

where $\bar{F}$ is the average inbreeding coefficient of the focal set. Additionally, Bermann et al. [49] calculated the relative increase in accuracy by adding phenotypic information as $\rho_{wp}^{-1}-1$, and the relative increase in accuracy by adding genomic information to the partial dataset as $\rho_{A,G}^{-1}-1$. Both formulas were multiplicated by 100 to express them in percentage.

We conducted two validation analyses to evaluate the different models’ predictive performance. The first assessed the ability to predict the breeding values of parents (for controlled mating), while the second evaluated the ability to predict the breeding values of individuals (for cloning).

The parent’s validation group consisted of parents without phenotyped progeny in the reduced dataset (validation set) but with phenotyped progeny in the full dataset (training set). Parent validation was performed under two scenarios: one involving selecting 100 parents and another selecting only 50 parents (Table 3).

Cross-validation for individuals involved progenies without phenotypic data in the reduced dataset (validation set) but with phenotypic data in the full dataset (training set). For the reduced dataset, the progeny test 5 was used (Table 4). Ideally, evaluations at 14 or 21 months, the target selection age, would have been preferable. For this reason, only one validation was performed.

The LR statistics were applied only to the genotyped individuals in all validations.

## 3. Results

### 3.1. Genetic Parameters

The narrow-sense heritability estimates obtained using the genomic model (ssGBLUP) were not significantly different from those using the pedigree-based model (ABLUP), except for DBH21 (Table 5). Heritability values ranged from 0.37 to 0.84 in the ssGBLUP model and from 0.33 to 0.86 in the ABLUP approach.

The genetic correlations among all traits were positive and ranged from moderate to high, with values between 0.51 and 0.97 across both models (Table 6). DBH showed the strongest correlations with all traits.

### 3.2. Model Comparison for Parent Selection

The LR statistics estimated for both models showed that the accuracy of the genomic-based models (ssGBLUP) was always higher than that of the pedigree-based models (ABLUP) in both validation scenarios, ranging from 0.37 to 0.66 with genomic information and 0.23 to 0.51 using only pedigree information (Table 7). The estimated bias was smaller with genomic information than without it in both validation scenarios, ranging from 0.08 to 0.27 and 0.21 to 0.36, respectively. When comparing the dispersion in both validation scenarios, using pedigree information in the first scenario showed slightly better dispersion for growth traits (TH and DBH) and nearly equal dispersion for heteroblasty (ADFO), ranging from 0.77 to 0.95. However, in the second validation scenario, the dispersion was better for models based on genomic information, with estimated values close to one, ranging from 0.82 to 0.95. The correlation between whole and partial dataset for ssGBLUP was always higher than for ABLUP in both validation scenarios, ranging from 0.52 to 0.77 and 0.28 to 0.58, respectively. The second validation showed the best values of accuracy, bias, dispersion, and correlation for ssGBLUP, making this the optimal scenario for validation.

The increase in accuracy by adding genomic information was higher than by adding phenotypes to the partial dataset (Table 8) for all traits, especially for growth traits (TH and DBH).

The LR statistics estimated for both models with MF showed that the accuracy with genomic information (ssGBLUP_MF) was always higher than using only pedigree information (ABLUP_MF) models in both validation scenarios, ranging from 0.36 to 0.67 and 0.21 to 0.48, respectively (Table 9). Both models exhibited bias in both validation scenarios, with ssGBLUP_MF displaying a greater range, from 0.15 to 1.6, compared to ABLUP_MF, which ranged from 0.04 to 0.43. When comparing the dispersion in both validation scenarios, ssGBLUP_MF showed values closer to one, ranging from 0.72 to 0.93, whereas for ABLUP_MF the range was from 0.66 to 0.91. The first validation showed practically equal dispersion values for all traits. However, in the second validation scenario, when only pedigree information with MF was used, the estimated dispersion was more variable. In contrast, with genetic information and MF, the estimates were closer to one. The correlation between whole and partial dataset was higher with ssGBLUP_MF that using ABLUP_MF, ranging from 0.57 to 0.80 and 0.29 to 0.58, respectively, in both validation scenarios. The second validation showed the best values of accuracy, bias, dispersion and correlation for ssGBLUP_MF, making this a better scenario for validation.

**Figure 1:**
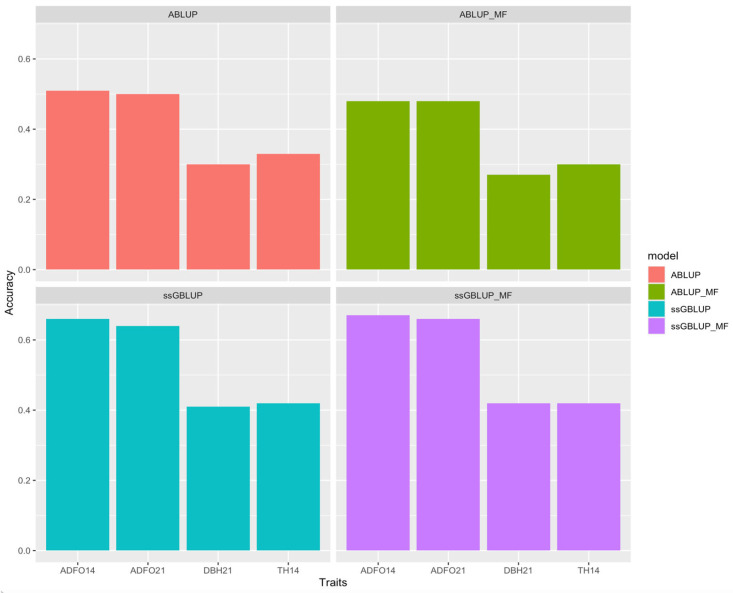
Accuracy of models for all traits in the second scenario of parent validation for ABLUP, ABLUP_MF, ssGBLUP, and ssGBLUP_MF models. ADFO14 and AFFO21: fraction of adult foliage in the canopy at 14 and 21 months; DBH21: diameter at breast height at 21 months; and TH14: total tree height at 14 months.

**Figure 2:**
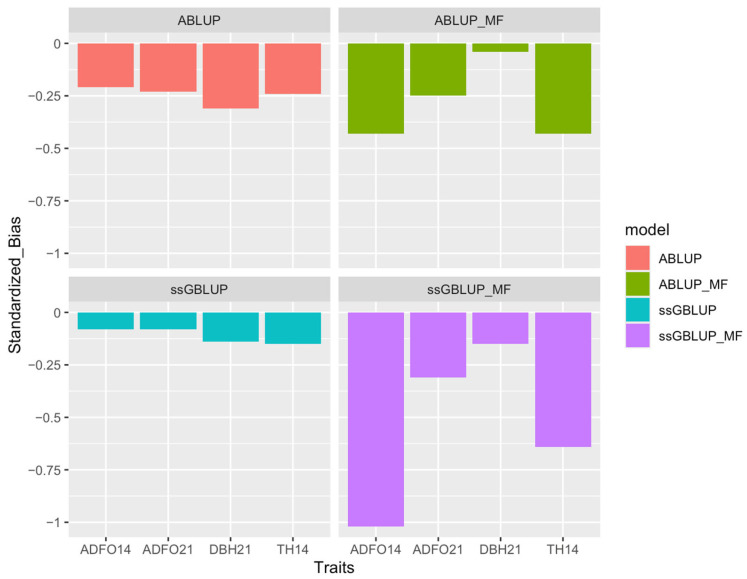
Bias of models for all traits in the second scenario of parent validation for ABLUP, ABLUP_MF, ssGBLUP, and ssGBLUP_MF models. ADFO14 and ADFO21: fraction of adult foliage in the canopy at 14 and 21 months; DBH21: diameter at breast height at 21 months; and TH14: total tree height at 14 months.

When comparing the four models, it is observed that the second validation scenario with the ssGBLUP model had the best predictive performance, with dispersion near one, relatively high accuracy (Figure 1), high correlation between whole and partial dataset and bias near zero (Figure 2).

### 3.3. Model Comparison for Individuals

The LR statistics estimated for both models showed that the accuracy of the genomic-based models (ssGBLUP) was always higher than that of the pedigree-based (ABLUP) models, ranging from 0.48 to 0.68 with genomic information and 0.37 to 0.53 using only pedigree information (Table 10). The estimated bias was near zero with both models, except for ADFO14 and ADFO21 using genomic information (ssGBLUP) and HT14 using ABLUP, ranging from 0.01 to 0.17 and 0.05 to 0.15, respectively. The dispersion using genomic information was slightly better for all traits (close to one) than using ABLUP, ranging from 0.90 to 1.09 and 0.83 to 1.20, respectively. The correlation between whole and partial dataset was higher with ssGBLUP that using ABLUP, ranging from 0.74 to 0.80 and 0.54 to 0.67, respectively.

The LR statistics estimated for both models with MF showed that the accuracy with genomic information (ssGBLUP_MF) was always higher than when using only pedigree information with MF (ABLUP_MF), ranging from 0.57 to 0.85 and 0.37 to 0.53, respectively (Table 11). The bias estimation was higher with genomic information than without, except for DBH, ranging from 0.04 to 1.28 and 0.36 to 0.93, respectively. When comparing the dispersion in both models, the model with genomic information and MF had estimates close to one. In contrast, the model with only pedigree information and MF exhibited more variable dispersions, ranging from 0.82 to 1.17. The correlation between whole and partial dataset was higher with ssGBLUP_MF (p(wp)) = 0.99) that using ABLUP_MF (p(wp)) = 0.60 to 0.69).

**Figure 3:**
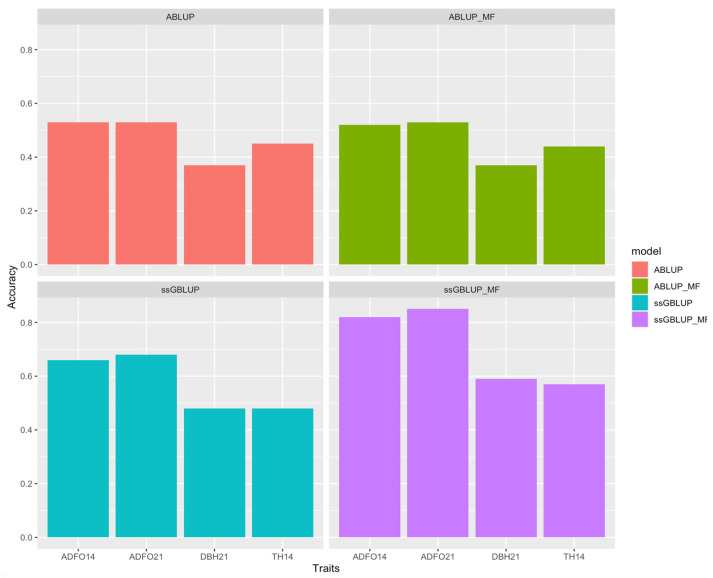
Accuracy of models for all traits in individual validation for ABLUP, ABLUP_MF, ssGBLUP, and ssGBLUP_MF models. ADFO14 and ADFO21: fraction of adult foliage in the canopy at 14 and 21 months; DBH21: diameter at breast height at 21 months; and TH14: total tree height at 14 months.

**Figure 4:**
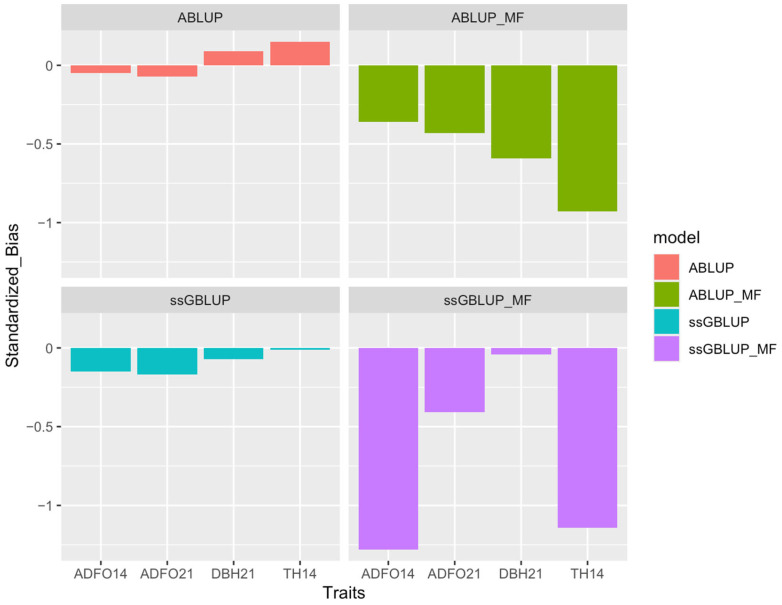
Bias of models for all traits in individual validation for ABLUP, ABLUP_MF, ssGBLUP, and ssGBLUP_MF models. ADFO14 and ADFO21: fraction of adult foliage in the canopy at 14 and 21 months; DBH21: diameter at breast height at 21 months; and TH14: total tree height at 14 months.

The statistics of predictive performance for the four models were better with the ssGBLUP model, with relatively high accuracy, except for ssGBLUP_MF (Figure 3), bias near zero (Figure 4), dispersion near one and high correlation between whole and partial dataset.

## 4. Discussion

Tree improvement programs globally are not very advanced due to the long generation cycles of even the fastest-growing species [50]. Tree selection based on genomic information has become an important tool in forest tree breeding [51]. Estimating genetic parameters plays a crucial role in the management of seed orchards and provides valuable guidance for developing the evaluation and selection strategy for the next generation of improvement [52,53]. In this study, the heritabilities estimated using ssGBLUP were consistently lower than those obtained from ABLUP across all evaluated traits. This discrepancy is consistent with findings in previous research and is often attributed to the increased resolution provided by genomic information, which enables more accurate partitioning of genetic and residual variances [10,54]. Marker-based relationship matrices capture Mendelian sampling and cryptic relationships that are not reflected in pedigree-based models, thereby refining the estimation of additive genetic variance and potentially reducing the estimated heritability [44,55]. In contrast, pedigree-based models may overestimate heritability by attributing unaccounted environmental or non-additive effects to additive genetic variance [56,57]. Genomic models, particularly those incorporating dense marker information, can disentangle these effects more accurately by capturing the realized proportion of shared alleles among individuals, thus providing a more precise estimate of additive genetic variance [44,55,58].

### 4.1. Predictive Model Performance

The effectiveness of genomic selection in breeding programs depends on the phenotypic quality and depth, the prediction model, the number and type of molecular markers, the size and composition of the training population, the accuracy of predicted genomic breeding values, and the relatedness between the training and validation populations [54,59]. Therefore, the optimal training population design is one of the most challenging aspects of GS. Beaulieu et al. [60] and Klápště et al. [52] underline the importance of the relationship between training and test populations in genomic selection models. The cross-validation approaches proposed by Legarra and Reverter [48], including “partial” and “whole” data based on differences in means, covariance, and correlation, are not usually implemented in tree breeding evaluations. In this study, the increase in accuracy obtained with ssGBLUP suggests that incorporating genomic information significantly influences the selection of superior individuals. Several studies have demonstrated the effectiveness of ssGBLUP in enhancing prediction accuracy for growth and wood quality traits in tree species [61,62,63,64]. By using realized genomic relationships rather than expected pedigree-based ones, ssGBLUP enhances the accuracy of breeding value predictions [10,44]. This improvement is especially relevant in populations with unbalanced pedigrees, where traditional models may not fully capture the true genetic variability [54]. The inclusion of genomic data allows for better discrimination among individuals with similar pedigree backgrounds but different genetic merits [65]. This enhanced resolution improves the estimation of breeding values, allowing for more precise selection of superior individuals and reducing the risk of selecting suboptimal candidates based solely on pedigree [44,54]. As a result, genomic selection contributes to increased selection accuracy and, consequently, a higher rate of genetic gain per unit of time or generation [66,67,68]. In our study, integrating genomic information led to increased accuracy and reduced bias in estimated breeding values (EBVs) compared to traditional pedigree-based models. Additionally, the dispersion of EBVs improved, with values approaching the ideal of one, indicating more accurate and consistent predictions of genetic merit. These findings align with previous studies that have reported similar improvements in accuracy through the inclusion of genomic data [40,52,64] and observed less biased predictions compared to ABLUP [63,69].

Metafounders are useful for characterizing relationships within and across populations by modeling the means and variances of unknown base population individuals [26,70]. For instance, Macedo et al. [71] demonstrated that metafounders enhanced the accuracy of genomic predictions by accounting for additional genetic variation that is not captured by traditional pedigree-based relationships. Similarly, Kudinov et al. [72] reported that incorporating metafounders in ssGBLUP models improved selection accuracy in both plant and animal breeding programs, particularly for traits with low heritability. Other reports demonstrated the advantages of metafounders in populations with complex or unknown pedigree structures [73,74,75]. However, in our study, except in the ssGBLUP model for individual selection, the inclusion of metafounders in both ssGBLUP and ABLUP models did not improve the prediction accuracy. These results agree with those of other studies, particularly in cases with well-connected populations or limited pedigree uncertainty [76,77,78].

The correlation between the full and partial datasets showed an improvement in stability for ssGBLUP compared to ABLUP, with considerable increases caused by the inclusion of genomic information. This trend is consistent with Berman et al. [49], who reported similar findings. In contrast, Callister et al. [78] found only a small improvement in stability between ABLUP and ssGBLUP. Including genomic information corrected the dispersion found for ABLUP models, particularly with the inclusion of MF. Similar results were obtained in other works [71,72,79], suggesting that MF could be an option for managing missing pedigree in ssGBLUP models. However, the inclusion of MF resulted in a significant increase in bias for all models, which could result from the relatively small number of genotyped individuals contributing to the estimation of gamma (γ), leading to a lack of connection between the genotyped individuals and the base population. Callister et al. [78] reported similar results when MF was included in the population. In contrast, in other studies, the bias was effectively eliminated with the inclusion of MF [71,79]. The study by Legarra et al. [26] underscores the importance of having sufficient representation within each metafounder group to ensure accurate estimation of the gamma matrix. Small or unbalanced groups can affect the stability and accuracy of relationship estimates, including those in the gamma matrix. In their 2024 study, Legarra et al. [80] further discuss the complexities of estimating gamma in highly unbalanced data, such as genotyped individuals far from base populations or many unknown parent groups within breeds. They propose maximum likelihood and pseudo-expectation–maximization methods to estimate gamma in these settings, emphasizing the need for careful consideration of group representation to avoid biased estimates. These results suggest that the metafounder approach needs to be tested with data that are more closely related to the base population and groups that have sufficient representation within each metafounder to improve the results.

After comparing different models with and without genomic information, including metafounders (MF) in both approaches, this study found that the best model to select parents for controlled matings and individuals for cloning is ssGBLUP without MF. The ssGBLUP accuracy for both validations (parents and individuals) ranged from 0.42 to 0.68, being lower for growth traits (TH and DBH) than for disease resistance (ADFO). These results confirm those obtained by Quezada et al. [40], encouraging the application of genomic prediction schemes for these traits in tree breeding programs. These results indicate that ssGBLUP models allow to predict BV with high accuracy, and thus to select parents for controlled matings and individuals for cloning with confidence, reducing the time and costs of implementing progeny trials.

### 4.2. Strategy of Selection

The adoption of GS in forest tree breeding allows for the shortening of breeding cycles, increased selection intensity, and improved accuracy of breeding values, all of which lead to increased genetic gains [81,82]. The findings from this study regarding growth and disease traits in *E. globulus* indicate moderate to high heritability for both traits. Therefore, the expected responses to selection for growth and disease resistance are favorable. Furthermore, the high age-age genetic correlations between ADFO at 14 months and ADFO at 21 months demonstrate the effectiveness of early selection, allowing selection at 14 months to predict performance at 21 months without a significant decrease in genetic gain. This reduces evaluation costs, and the time required to obtain improved individuals. Early selection is a cornerstone of modern tree breeding programs, significantly improving their efficiency and effectiveness [1,8]. By identifying superior genotypes at juvenile stages, breeders can reduce generation intervals, increase selection intensity, and improve overall genetic gains [83,84].

**Figure 5:**
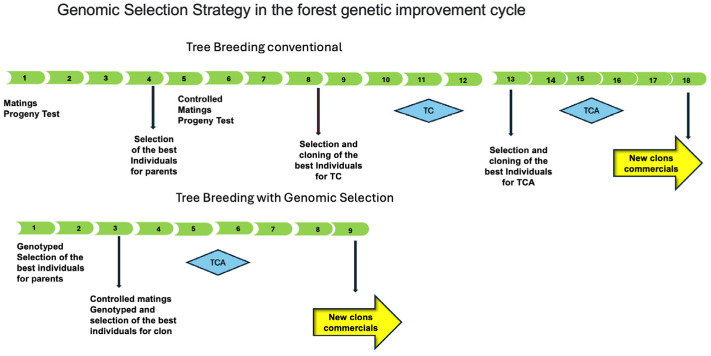
Conventional breeding cycle vs. breeding cycle with the inclusion of genomic selection for obtaining commercial clones of *Eucalyptus globulus*, the numbers correspond to the cycle years.

Additionally, advancements in genomic selection have enhanced early selection methods by enabling accurate prediction of mature phenotypes from young plants, thus facilitating more precise and efficient breeding decisions [85]. The findings reported by this study suggest that two stages of selection are recommended in tree breeding programs aimed at selecting commercial clones. The first stage involves selecting the best parents for controlled matings. The second stage focuses on selecting the best progenies for cloning and their evaluation in clonal tests (CT), which evaluates many genotypes with few replicates, and amplified clonal testing (ACT), which evaluates fewer genotypes with more replicates (Figure 5). Including GS in both stages would reduce the breeding cycle by eliminating progeny testing and the first type of CT, thus reducing implementation costs and increasing genetic gains. The ssGBLUP model evaluated in this study would function at two levels: initially, by selecting the best parents in the first year of the forest genetic improvement cycle, and subsequently, by identifying the best individuals for cloning in the third year.

## 5. Conclusions

The implementation of GS demonstrated that it is a useful and straightforward tool for selection of candidates. While ABLUP relies exclusively on pedigree information and may overestimate additive genetic variance, ssGBLUP incorporates genomic relationships, leading to more accurate and less biased predictions of breeding values. However, the inclusion of metafounders (MF) increased bias, suggesting that MF should be tested using data more representative of the base population to achieve more reliable outcomes. Therefore, genomic selection (GS) can be effectively integrated at two key stages of the breeding cycle: the selection of superior parents for controlled matings, and the early selection of individuals in the nursery stage, potentially reducing the need for extensive field evaluation of selected clones.

## Figures and Tables

**Table 1:** Summary of phenotypic and genotypic data across all trials. The table includes the installation year, tree age at measurement, mating type (open pollination, OP; or controlled mating, CM), number of trees phenotyped, and number of trees genotyped for each trial. Progeny tests from OP represent half-sib families, while those from CM represent full-sib families.

Trials	Installation Year	Age (Year)	Matting Type	Number of Trees Phenotyped	Number of Trees Genotyped
Parents (seed orchard first generation)	1996	28	OP	604	80
Parents (seed orchard second generation)	2002	22	OP	455	446
Progeny test 1	2011	13	OP	3756	975
Progeny test 2	2014	10	CM	551	87
Progeny test 3	2015	9	CM	644	108
Progeny test 4	2016	8	CM	989	194
Progeny test 5	2017	7	CM	1591	199
Progeny test 6	2019	5	CM	2276	320
Total				10,373	2409

**Table 2:** Distribution of trees across adult foliage (ADFO) categories at two time points: 14 months (ADFO14) and 21 months (ADFO21). Category 1 = no adult foliage, Category 2 = up to 50% adult foliage, and Category 3 = more than 50% adult foliage. The number of trees per category is shown for each trait.

Trait	Number of Trees	Category
ADFO14	6145	1
ADFO14	3565	2
ADFO14	192	3
ADFO21	2139	1
ADFO21	5658	2
ADFO21	1828	3

**Table 3:** Validation design for parent selection under two scenarios (V1 and V2). Each scenario includes the number of phenotypic and genotypic records in the training and validation sets, the progeny tests included in training, and the number of parents evaluated in validation. Progeny tests are labeled from 1 to 6.

	Set Training			Set Validation		
	Phenotypes	Genotypes	Number of Progeny Test	Phenotypes	Genotypes	Parents
V1	9832	2359	1, 2, 3, 4, 5, 6	2121	478	100
V2	9832	2359	1, 2, 3, 4, 5, 6	1329	272	50

**Table 4:** Validation design for the selection of individuals for cloning. The table shows the number of phenotypic and genotypic records in the training and validation sets, along with the progeny tests included in each. Progeny tests 1 to 4 were used for training, while progeny test 5 was used for validation.

Set Training			Set Validation		
Phenotypes	Genotypes	Number of Progeny Test	Phenotypes	Genotypes	Number of Progeny Test
5965	2359	1, 2, 3, 4	1591	199	5

**Table 5:** Narrow-sense heritability estimates (h^2^) and 95% high posterior density (HPD) intervals for four traits in *Eucalyptus globulus* under the ssGBLUP and ABLUP models. Traits include total tree height at 14 months (TH14), proportion of adult foliage at 14 and 21 months (ADFO14 and ADFO21), and diameter at breast height at 21 months (DBH21).

	ssGBLUP		ABLUP	
Trait	h^2^	HPD Interval (95%)	h^2^	HPD Interval (95%)
TH14	0.37	0.31–0.43	0.33	0.26–0.40
ADFO14	0.84	0.79–0.89	0.84	0.77–0.88
DBH21	0.53	0.47–0.58	0.65	0.62–0.69
ADFO21	0.83	0.78–0.88	0.86	0.80–0.90

**Table 6:** Genetic correlations (rg) among traits in *Eucalyptus globulus* estimated using ssGBLUP (above the diagonal) and ABLUP (below the diagonal) models. Values in parentheses indicate the 95% high posterior density (HPD) intervals. Traits include total tree height at 14 months (TH14), proportion of adult foliage at 14 and 21 months (ADFO14, ADFO21), and diameter at breast height at 21 months (DBH21).

rg	TH14	ADFO14	DBH21	ADFO21
TH14	1	0.52 (0.44–0.60)	0.83 (0.79–0.86)	0.51 (0.44–0.59)
ADFO14	0.64 (0.56–0.71)	1	0.66 (0.60–0.72)	0.97 (0.95–0.98)
DBH21	0.77 (0.73–0.81)	0.69 (0.64–0.75)	1	0.70 (0.64–0.75)
ADFO21	0.61 (0.53–0.70)	0.97 (0.95–0.99)	0.73 (0.67–0.78)	1

**Table 7:** Linear regression statistics for the ssGBLUP and ABLUP models under two validation scenarios (V1 and V2) for selected parents. Reported metrics include accuracy, standardized bias, dispersion, and the correlation between whole and partial dataset (p(wp)). Traits include total tree height at 14 months (TH14), proportion of adult foliage at 14 and 21 months (ADFO14, ADFO21), and diameter at breast height at 21 months (DBH21).

		ssGBLUP				ABLUP			
Traits	Validation	Accuracy	Standardized Bias	Dispersion	p(wp)	Accuracy	Standardized Bias	Dispersion	p(wp)
TH14	V1	0.37	−0.24	0.72	0.59	0.27	−0.26	0.77	0.43
ADFO14	V1	0.61	−0.16	0.94	0.72	0.43	−0.29	0.95	0.50
DBH21	V1	0.37	−0.27	0.73	0.52	0.23	−0.36	0.77	0.28
ADFO21	V1	0.59	−0.17	0.90	0.70	0.43	−0.29	0.90	0.49
TH14	V2	0.42	−0.15	0.88	0.69	0.33	−0.24	0.73	0.51
ADFO14	V2	0.66	−0.08	0.95	0.77	0.51	−0.21	0.90	0.58
DBH21	V2	0.41	−0.14	0.82	0.60	0.30	−0.31	0.80	0.37
ADFO21	V2	0.64	−0.08	0.92	0.75	0.50	−0.23	0.86	0.57

**Table 8:** Relative increase in prediction accuracy (%) for the second validation scenario when adding phenotypic (Inc Phen%) and genotypic (Inc Geno%) information to the partial dataset. Traits include total tree height at 14 months (TH14), proportion of adult foliage at 14 and 21 months (ADFO14, ADFO21), and diameter at breast height at 21 months (DBH21).

Traits	Inc Phen%	Inc Geno%
TH14	45	96
ADFO14	30	61
DBH21	67	194
ADFO21	33	59

**Table 9:** Linear regression statistics for the ssGBLUP_MF and ABLUP_MF models under two validation scenarios (V1 and V2) for selected parents. Metrics include prediction accuracy, standardized bias, dispersion, and the correlation between whole and partial dataset (p(wp)). Traits evaluated are total tree height at 14 months (TH14), proportion of adult foliage at 14 and 21 months (ADFO14, ADFO21), and diameter at breast height at 21 months (DBH21).

		ssGBLUP_MF				ABLUP_MF			
Traits	Validation	Accuracy	Standardized Bias	Dispersion	p(wp)	Accuracy	Standardized Bias	Dispersion	p(wp)
TH14	V1	0.36	−1.60	0.72	0.63	0.24	−0.35	0.72	0.40
ADFO14	V1	0.61	−1.14	0.93	0.76	0.40	0.18	0.91	0.49
DBH21	V1	0.39	−0.87	0.72	0.57	0.21	−0.12	0.73	0.29
ADFO21	V1	0.60	−0.72	0.89	0.74	0.40	0.14	0.88	0.48
TH14	V2	0.42	−0.64	0.81	0.71	0.30	−0.43	0.66	0.47
ADFO14	V2	0.67	−1.02	0.93	0.80	0.48	−0.43	0.85	0.58
DBH21	V2	0.42	−0.15	0.76	0.63	0.27	−0.04	0.73	0.38
ADFO21	V2	0.66	−0.31	0.88	0.78	0.48	−0.25	0.82	0.57

**Table 10:** Linear regression statistics for the ssGBLUP and ABLUP models used in the selection of individual trees for cloning. Reported metrics include prediction accuracy, standardized bias, dispersion, and the correlation between whole and partial dataset (p(wp)). Traits include total tree height at 14 months (TH14), proportion of adult foliage at 14 and 21 months (ADFO14, ADFO21), and diameter at breast height at 21 months (DBH21).

		ssGBLUP			ABLUP			
Traits	Accuracy	Standardized Bias	Dispersion	p(wp)	Accuracy	Standardized Bias	Dispersion	p(wp)
HT14	0.48	−0.01	0.90	0.74	0.45	0.15	0.83	0.66
ADFO14	0.66	−0.15	1.08	0.80	0.53	−0.05	1.16	0.67
DBH21	0.48	−0.07	0.94	0.74	0.37	0.09	0.99	0.54
ADFO21	0.68	−0.17	1.09	0.80	0.53	−0.07	1.20	0.67

**Table 11:** Linear regression statistics for the ssGBLUP_MF and ABLUP_MF models using a validation method for the selection of individual trees. Metrics reported include prediction accuracy, standardized bias, dispersion, and the correlation between whole and partial dataset (p(wp)). Traits evaluated are total tree height at 14 months (TH14), proportion of adult foliage at 14 and 21 months (ADFO14, ADFO21), and diameter at breast height at 21 months (DBH21).

	ssGBLUP_MF				ABLUP_MF			
Traits	Accuracy	Standardized Bias	Dispersion	p(wp)	Accuracy	Standardized Bias	Dispersion	p(wp)
HT14	0.57	−1.14	1.005	0.99	0.44	−0.93	0.82	0.69
ADFO14	0.82	−1.28	1.007	0.99	0.52	−0.36	1.14	0.68
DBH21	0.59	−0.04	1.004	0.99	0.37	−0.59	0.97	0.60
ADFO21	0.85	−0.41	1	0.99	0.53	−0.43	1.17	0.69

## Data Availability

The data presented in this study are available on request from the corresponding author due to privacy.
